# Carotid Artery Stenting Intervention to Enhance Global Brain Blood Flow and Cognition in Carotid Artery Disease: Preliminary Findings from a Prospective Follow-Up MRI Study

**DOI:** 10.3390/medicina61050848

**Published:** 2025-05-05

**Authors:** Raminder Kaur, Paul Summers, William Siu, George Medvedev, Sam M. Doesburg, Xiaowei Song

**Affiliations:** 1 Research and Evaluation, Fraser Health Authority, Surrey, BC V3T 0H1, Canada; raminder.kaur1@fraserhealth.ca; 2 Biomedical Physiology & Kinesiology, Simon Fraser University, Burnaby, BC V5A 1S6, Canada; sam_doesburg@sfu.ca; 3 SFU ImageTech Lab, Surrey Memorial Hospital, 13750 96 Avenue, Surrey, BC V3V 1Z2, Canada; paul_summers@sfu.ca; 4 Medical Imaging, Royal Columbian Hospital, New Westminster, BC V3L 3W7, Canada; 5 Division of Neurology, Royal Columbian Hospital, New Westminster, BC V3L 3W7, Canada; george.medvedev@fraserhealth.ca

**Keywords:** carotid artery stenosis, magnetic resonance imaging, perfusion, cerebrovascular disease, intervention, aging, brain health, stroke, cognitive decline, prevention

## Abstract

*Background and Objectives*: The benefit of carotid artery stenting (CAS) for stroke prevention has been established, but less is known about CAS’s effect on cognition. Here, we investigate (1) changes in the blood flow in both treated and non-treated carotid arteries, (2) associations between the severity of artery occlusion and CAS-induced flow change, and (3) whether the flow changes relate to cognitive improvement. *Materials and Methods*: We used quantitative flow magnetic resonance imaging to assess blood flow and computerized neurocognitive assessment to evaluate cognitive performance. Fourteen patients identified for CAS as part of their standard care participated in this study; ten completed the CAS procedure and the pre- and post-CAS assessments (age = 77.0 ± 5.6; 70% males). *Results*: An increased ipsilateral flow following CAS was seen in 70% of the participants, while 50% also showed an increase in the total flow. The participants with ≥90% stenosis showed the greatest flow changes, such that the post-CAS flow was 60% higher relative to pre-CAS (*p* < 0.050). Cognitive responses to the flow increase were variable: attention showed a positive association; in comparison, higher cognitive flexibility and memory were only seen when treated stenosis was below 80%. *Conclusions*: Our preliminary findings highlight the impact of CAS and the complex relationship between blood flow and cognitive changes post-CAS, warranting larger-scale studies with extended follow-up periods.

## 1. Introduction

Approximately one-fifth of all acute ischemic strokes are associated with carotid artery stenosis, and in cases of severe (≥70%) stenosis, ipsilateral strokes recur in up to 30% of patients [1,2]. Both carotid artery stenting (CAS) and endarterectomy (CEA) minimize the future risk of cerebral stroke and potentially improve cognitive functioning [2]. Although CEA is still the gold standard for severe stenosis, CAS has emerged as a less invasive alternative [1,2,3]. While CAS is well-documented as safe and effective, its impact on blood flow dynamics and cognitive correlates remains underexplored [4,5,6]. Beyond its established role in reducing stroke risk, CAS’s ability to normalize cerebral blood flow may offer important cognitive benefits, warranting further exploration.

Cognitive function is increasingly recognized as a crucial health outcome that impacts daily living and quality of life [7]. In the context of carotid artery stenosis, where reduced cerebral blood flow may contribute to cognitive decline, interventions such as CAS that aim to restore perfusion have the potential to alleviate dysfunction caused by chronic hypoperfusion [8,9]. However, as a research focus for only a decade, investigations into how CAS affects cognition remain limited and have yielded mixed findings. An earlier review based solely on pre- and post-operative neuropsychological assessments suggested that CAS (as CEA) did not significantly affect neurocognitive outcomes [10]. In contrast, several recent MRI studies suggest cognitive changes following CAS [2,4,11], though findings remain inconsistent across the literature. For example, a study reported sustained cognitive improvements six months following CAS that lasted over three years in patients with cerebral lacunar infarction and severe internal carotid artery (ICA) stenosis [12]. Other studies reported both improvement and deterioration in certain cognitive outcomes [9] or no insignificant changes after angioplasty with stenting [8]. These inconsistencies highlight the need for further research to clarify the cognitive effects of CAS, particularly using imaging and standardized cognitive assessments in well-characterized patient cohorts.

Here, we conducted a prospective follow-up study to better understand the role of carotid revascularization in enhancing blood flow in carotid artery disease. We applied quantitative flow MRI (QFlow), a particularly useful technique in vascular cardio/cerebrovascular imaging, to measure blood flow dynamics in patient participants before and after the CAS procedure. Given the relevance of vascular topography, in particular, the middle cerebral artery (MCA) in acute ischemic stroke and prognosis [13,14], we incorporated MCA characteristics in the analysis. We also examined the relationship between quantitative flow changes and neurocognitive changes using Central Nervous System Vital Signs (CNSVS). Our specific objectives were to investigate (1) changes in the blood flow in both treated and non-treated carotid arteries, (2) associations between the severity of artery narrowing and CAS-induced flow change, and (3) whether the flow changes relate to cognitive improvement.

## 2. Materials and Methods

### 2.1. Study Design, Participant, and Clinical Care with CAS

**Figure 1:**
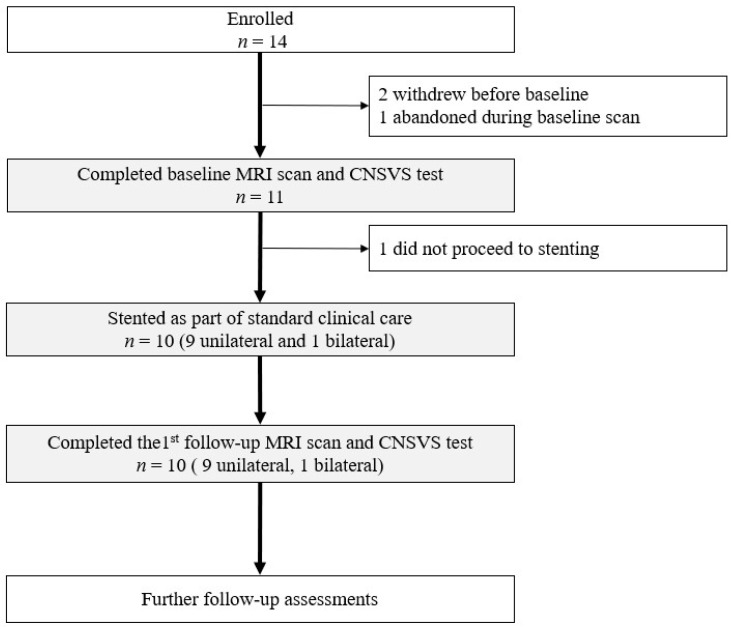
Flowchart showing the study sample. Data from the carotid arterial stenosis patient participants who had a carotid artery stenting procedure and completed both baseline and the first follow-up MRI scans were used in the analysis, as highlighted using gray boxes. Note: “*n*” represents number of participants; CNSVS represents Cognitive Nervous System Vital Signs.

This study used a pre–post design in a purposive cohort of patients with severe carotid stenosis (i.e., ≥70% occlusion). Male and female patients scheduled for CAS between May 2022 and December 2024, as part of their standard care, were considered for participation. The study design involved three patient visits to the MRI lab: one before CAS and follow-ups at two and four months post-CAS. CAS was performed within two weeks of baseline MRI by an experienced interventional radiologist (i.e., coauthor W.S.). Follow-ups were performed within one week of the target time point (five weeks from baseline). Each visit included an MRI examination and neuropsychological testing as detailed below (Figure 1). The participants who completed the CAS procedure and both baseline and first follow-up MRI scans were used in the present analysis (Figure 1).

CAS was performed under clinical standard care. All participants had chronic, gradually progressive carotid stenosis, typically developing over several years. The decision to proceed with CAS was made based on established clinical guidelines, incorporating imaging evidence of vascular anatomy and stenosis severity, and individualized risk–benefit assessments. This process involved a multidisciplinary team—including neurologists, stroke specialists, interventional neuroradiologists, vascular surgeons, cardiologists, and primary care physicians—as well as shared decision-making with the patient and their caregivers. Each intervention took approximately 30–40 min to complete. No general anesthesia or sedation was given for these CAS procedures. Preparation and recovery from local anesthesia in the operation room added 30–60 min. All participants were stable post-procedure without any complications, such as stroke, transient ischemic attack, bradycardia, or hypotension. Each participant was allowed four weeks to recover fully from the procedure prior to the follow-up MRI scan, minimizing possible acute effects of the CAS procedure.

This study received approval from the Harmonized Approval involving Fraser Health Authority and Simon Fraser University Research Ethics Boards (H18-02495; FHA2018-058), with informed written consent signed by each participant before the initiation of the MRI experiment or any other research data collection.

### 2.2. Stenosis Quantification and Vascular Configuration

Pre- and post-CAS digital subtraction angiography was used to derive the degree of carotid stenosis at each time point. The baseline evaluation formed the basis of recruitment, with each participant having at least one severely stenosed internal carotid artery (ICA). The degree of stenosis was assessed using the NASCET formalism [15] and recorded for those vessels having severe (≥70%) stenosis with a precision of 5%. As clinical practice considers stenoses <70% to be functionally normal, the degree of stenoses in these vessels was not recorded in the clinical notes [16].

As possible confounding effects, the angiographic examinations were also reviewed for the presence of an isolated middle cerebral artery (MCA; i.e., deficiencies in the Circle of Willis) and possible tandem lesions. With an isolated MCA, the ipsilateral A1 segment of the anterior cerebral artery and the ipsilateral posterior communicating artery are hypoplastic (either tiny or absent). This results in the MCA being supplied only by the ipsilateral ICA, as no blood flow from the contralateral ICA or the basilar artery can arrive through the anterior cerebral artery or posterior cerebral artery, respectively. In these patients, the impact of stenting on contralateral and total flow may differ from that in patients having an intact Circle of Willis. A possible tandem lesion, i.e., one vessel having a second stenosis, was also assessed, in which the carotid bifurcation stenosis may not dominate flow limitation and thus limit the impact of stenting.

### 2.3. MRI Acquisition

The MRI examinations were conducted on a Philips Ingenia 3.0 Tesla CX system with a 32-channel head coil (Philips Healthcare, Best, The Netherlands). These examinations included acquisitions for T1-weighted structural imaging, task-state functional MRI, perfusion mapping with pseudo-continuous arterial spin, diffusion-weighted imaging, time-of-flight carotid angiography, and QFlow with the 2D phase-contrast technique, which was considered in this study.

**Figure 2:**
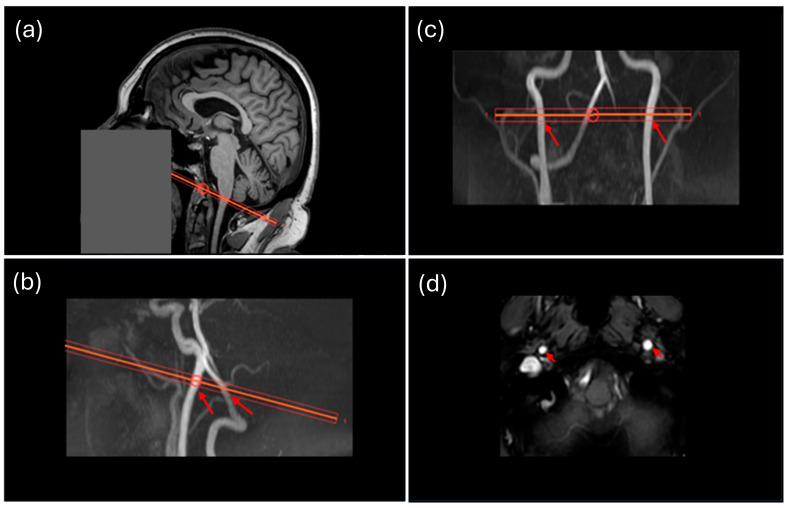
Example MRI acquisition and processing for flow quantification. (**a**) Sagittal view showing the oblique plane of the imaging placement across the ICAs (orange line, with a circle showing the central point), overlaying on the T1-weighted anatomical image. (**b**) Sagittal view showing the oblique plane placement of the imaging placement (orange line) crossing the bilateral ICAs (red arrows). (**c**) Coronal view showing the imaging placement (orange line) crossing the bilateral ICAs (red arrows). (**d**) Axial view highlighting the bilateral ICAs. Red arrows indicate the bilateral ICAs. Regions of interest (ROIs) of the two ICAs were drawn on this plane for flow velocity calculation within the ICAs.

QFlow assessment based on MRI phase-contrast imaging is a non-invasive method used to measure blood flow velocity and volume through vessels like carotid arteries (Figure 2). The technique employs through-plane 2D velocity mapping to calculate the mean blood flow over the cardiac cycle, providing quantitative insights into ICA flow [17]. During data acquisition, a left index finger-mounted sensor was used to monitor pulse wave dynamics for every participant. This plethysmography information was used for cardiac synchronization to reconstruct flow velocity of cardiac phases, inferring individual blood pressure waves while blood moved through the arteries. Slices for the QFlow MRI acquisition were oriented as close to perpendicular as possible to straight longitudinal sections of both ICAs. Scanning parameters included VENC 90 cm/s, flip angle 10°, TE/TR 5.3/9.0 ms, FOV 150 × 100 mm, 5 mm slice thickness, and 256 reconstruction matrix. A 60 × 60 median filter was used for background correction.

### 2.4. Flow Quantification from Flow MRI

The through-plane 2D velocity maps were used to calculate mean blood flow over the 15 frames of the cardiac cycle as follows: Two raters independently drew regions of interest (ROIs)—one based on the vessel hyperintensity within the signal magnitude image and the other on the peak forward-going speed in the angiographic image derived from the flow acquisition. ROIs were drawn for both the right and left ICA using image display and interaction tools (Rater 1 with ITK-SNAP © version 3.8.0 (ITK-SNAP 3.x Team, University of Pennsylvania, Philadelphia, PA, USA); Rater 2 with FSLview version 3.2.0 (FMRIB Software Library, University of Oxford, Oxford, UK). The mean velocity across each ROI over the entire cardiac cycle was calculated using FSLstats, from FSL version 6.2.0, and the mean flow through the vessel’s cross-section was calculated using the equation:

$$Flow\;\left({cm}^{3}/min\right)=Mean\;Velocity\;\left(cm/s\right)\times ROI\;Area\;\left({mm}^{2}\right)\times \frac{60\;(s/min)}{100\;({mm}^{2}/{cm}^{2})}$$

To assess inter-rater reliability, we performed a Bland–Altman analysis [18] to compare flow measurements between raters. Additionally, we calculated Dice similarity coefficients (Dice scores) to quantify the spatial overlap between regions of interest (ROIs) across raters. Based on the findings, a ±20% relative change in flow was adopted to distinguish physiologically meaningful changes from measurement variability. Errors in flow analysis may occur due to human errors if aliasing of the voxel-wise velocity estimate occurs due to a blood velocity that exceeds the velocity encoding range or if background phase differences in the acquired images lead to biasing of the velocity field. The velocity maps were inspected for aliasing in the ICA segment and residual background phase that could lead to errors. As none were observed (the above-mentioned filter minimizes background bias), mathematical corrections were not needed. 

### 2.5. Neuropsychological Testing

The CNSVS is a valid computer-based test battery that assesses various domains of neurocognitive processes [19]. These include memory of composite, verbal, and visual types, motor, psychomotor, and processing speed measures, attention of complex and simple forms, cognitive flexibility, and executive function. Depending on the domain, the CNSVS generates various raw scores, such as correct responses, taps, omission and commission errors, and reaction times. These were used to produce domain-specific scores. The raw cognitive domain scores were standardized for age (mean = 100, sd = 15). The Neurocognitive Index (NCI) was calculated as the mean of the domain scores [19].

### 2.6. Statistical Evaluation

Descriptive statistics were used to compare the demographic and other baseline characteristics, including comorbidity, medication, and lifestyle factors. Continuous variables were tested using the paired *t*-test, and discrete variables were tested using the Chi-square test. For each participant, flow measurements were obtained for each ICA at each time point. At each time point, the total flow was calculated as the sum of the ipsi- and conter-lateral ICA flows. The pairwise flow changes between time points were calculated in both absolute (i.e., follow-up − baseline values) and relative forms (i.e., 100 × (follow-up − baseline)/baseline values). To assess the lateralization of flow, we calculated the difference between right and left ICA flows (Right ICA − Left ICA). We examined the relationship between flow and the degree of stenosis, both before and after CAS, in the treated and non-treated sides using Spearman correlation and linear and exponential regression analyses. Additionally, we compared flow changes for those with or without an isolated MCA.

The absolute and relevant pairwise pre- vs. post-CAS differences in the cognitive testing scores were examined for the participants using paired-samples *t*-tests. The measures were further grouped based on the ICA stenosis level of the treated side into three groups (i.e., 70% group: 70–79% stenosis; 80% group: 80–89% stenosis, and 90% group: ≥90% stenosis). Relationships between the relative changes in the flow and cognitive testing scores were examined at different stenosis levels.

Statistical analyses and result visualizations were performed using codes developed with Matlab 2022b (The MathWorks, Inc., Natick, MA, USA). The significance level was set at *p* = 0.050.

## 3. Results

### 3.1. Participant Demographics and Characteristics

Of the enrolled participants, 10 completed CAS and both the pre- and post-CAS assessments (age range: 69–86, mean = 77.0 ± 5.6 years; 70% males; Figure 1; Table 1). Six (i.e., 60%) of the participants had an isolated MCA (where the MCA only received flow from the ipsilateral ICA without additional contribution from the Circle of Willis via the anterior or posterior communicating arteries). Nine of the participants underwent unilateral CAS (six left; three right), while the other participants had bilateral CAS (i.e., one left and one right). Among these 11 stented vessels analyzed for flow, 2 were in the 70% stenosis group, 5 in the 80% group, and 4 in the 90% group (Table 2).

### 3.2. Inter-Rater Reliability of Flow Quantification

The Bland–Altman analysis confirmed strong inter-rater reliability, with a mean bias of 6.06 ± 11.51 mL/min (Supplementary Figure S1). A Dice coefficient of 0.88 ± 0.07 indicated high overlap consistency in the ROI definition (Supplementary Figure S1). These results ensured reliable flow measurements. Based on the 95% CI of the agreement between readers and the mean flows rounded down two standard deviations, a percentage change (i.e., 20%) was used to represent the limits of sensitivity for a measurable change in flow. Small flow changes were considered marginal (Supplementary Figure S1)

### 3.3. Objective 1: Flow Changes in the Treated vs. Non-Treated ICAs

**Figure 3:**
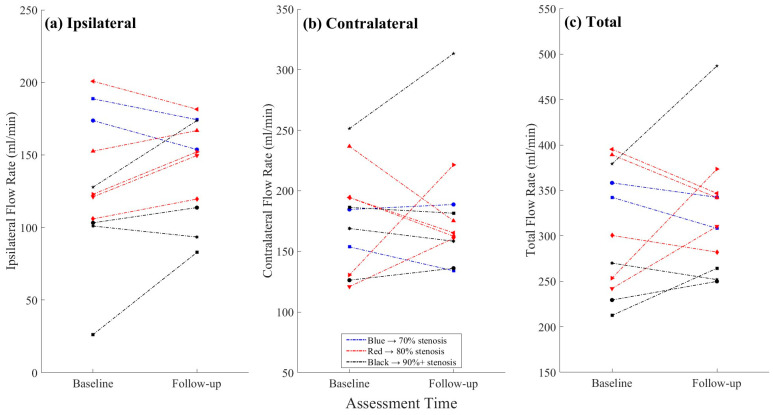
Individual changes in internal carotid artery (ICA) flow from baseline to follow-up. (**a**) Ipsilateral ICA flow; (**b**) Contralateral ICA flow; (**c**) Total ICA flow (sum of ipsilateral and contralateral). Note: Participants are color-coded by baseline stenosis severity group (blue = 70%, red = 80%, black = 90%); individuals are presented using unique marker shapes. Lines connecting data points in panels (**a**–**c**) are also color-coded by stenosis severity, as shown in the legend in panel (**b**).

The mean ipsilateral ICA flow changed from 129.4 ± 48.9 mL/min at baseline to 141.9 ± 34.2 mL/min at follow-up (Table 1), corresponding to an average increase of 26.9% (±64.8%). While this change was not statistically significant at the group level, stratifying by baseline stenosis revealed informative results (see Section 3.4). A post-CAS flow increase in the ipsilateral ICA was observed in seven participants, including five with a measurable increase (i.e., ≥20%, including one with 216% increase) and two with a marginal rise to 12.9%, while the other three participants showed a marginal flow reduction of up to 11.9% (Table 2; Figure 3).

The contralateral ICA flow changed from 177.1 ± 42.8 mL/min at baseline to 181.6 ± 49.9 mL/min at follow-up, amounting to a 5% (±27.7%) increase, on average. An increase in the contralateral ICA flow was seen in four participants, including two with an isolated MCA. The other four participants with an isolated MCA showed up to a 26% decrease in the contralateral ICA flow, while the decrease was up to 15% in the MCA-normal participants.

### 3.4. Objective 2: ICA Flow Changes by Stenosis Severity

**Figure 4:**
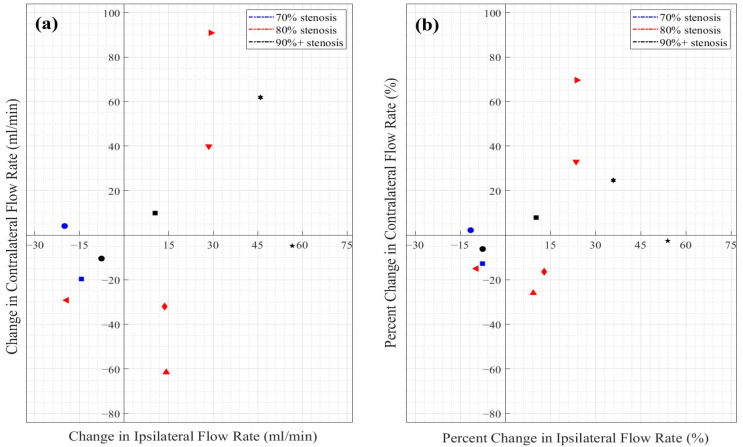
Comparison of changes in ipsilateral and contralateral flows between baseline and follow-up scans. (**a**) Absolute change in flow rate (mL/min). (**b**) Relative change in flow rate (%). Note: Each marker represents an individual participant. Stenosis groups are shown in different colors (blue: 70%, red: 80%, black: ≥90%); each unique symbol corresponds to one individual. Axes are centered at the origin to highlight directional changes in flow. One subject exhibited an extraordinary relative increase in ipsilateral flow (216%) and is scaled down by a factor of 1/4 for visualization clarity on the axis scale in (**b**).

Most of the participants in the 80% and 90% stenosis groups exhibited stable or increased ipsilateral flow following CAS (Figure 3a). The participants with 70% stenosis demonstrated a slight decrease in the total ICA flow. In contrast, the participants in the 80% stenosis exhibited a small increase (mean: +9.0 ± 27.3 mL/min), driven by improvements in both the ipsilateral (+12.0 ± 13.7 mL/min) and contralateral (+9.0 ± 41.0 mL/min) arteries. The most substantial flow changes were observed in the 90% stenosis group, where the total ICA flow increased by +13.8 ± 16.0 mL/min (Figure 3). This was primarily due to a pronounced rise in the ipsilateral flow (63.7 ± 103.1 mL/min, i.e., a 63.6% relative increase on average, with considerable individual variability), while the contralateral flow showed a more modest increase (+5.9 ± 13.8 mL/min; Figure 3). Despite a seemingly positive association between the ipsilateral and contralateral flows, no significant correlation was found in either the absolute (Figure 4a) or relative (Figure 4b) changes between the two sites (correlation coefficient r’s < 0.47; p’s > 0.150). The variability in the percent change, particularly on the ipsilateral side due to one outlier (216%), is further visualized (Figure 4b).

### 3.5. Objective 3: Cognitive Changes in Relation to Flow Changes

**Figure 5:**
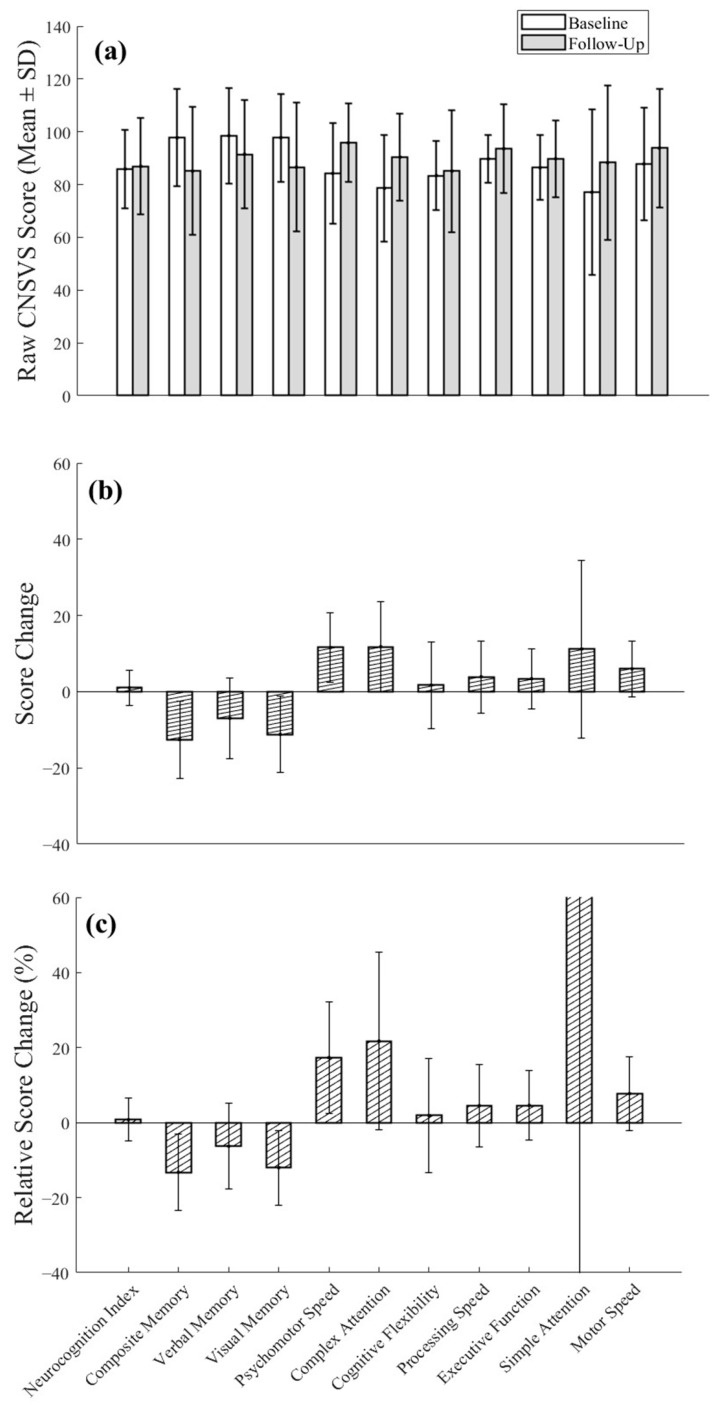
Group mean differences in cognitive scores with 95% confidence intervals. (**a**) Assessment scores pre-CAS (open bars) and post-CAS (filled bars). (**b**) Mean values of the absolute changes in the normalized scores for subject-wise differences between baseline and follow-up, where 100 represents the general population or group average. Lower scores indicate poorer performance. (**c**) Relative change in normalized scores. Note: Bars represent group means, with error bars indicating 95% confidence intervals. One outlier value (184.2%) for the simple attention domain of the 90% stenosis group is truncated on the axis scale for visualization clarity in (**c**).

While most cognitive domains showed numerical improvement, visual memory demonstrated a significant decrease (t(8) = –2.58, *p* = 0.032, two-tailed). Composite memory also showed a trend of reduction (*p* = 0.067). All other domains showed non-significant changes (*p* > 0.050), despite some domains (e.g., attention, motor speed) exhibiting relatively large mean differences (Figure 5). In our sample, age did not differ by baseline stenosis level (*p* = 0.871). Furthermore, age was not correlated with cognitive performance (the correlation coefficient ranged from r = −0.51 (*p* = 0.141) for processing speed to r < −0.20 (*p* = 0.649) for executive function) or with the change in cognitive scores (e.g., r = −0.63 for composite memory vs. age as the highest, *p* = 0.053).

**Figure 6:**
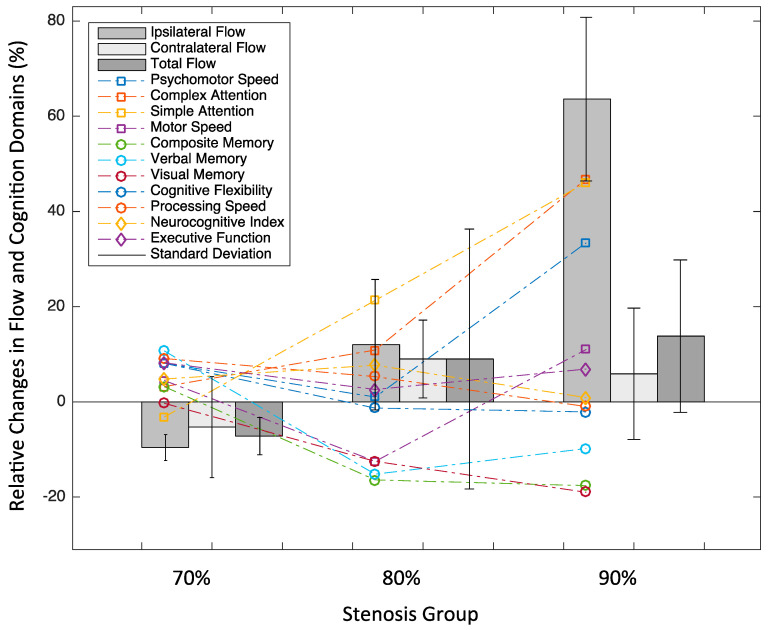
Post-CAS changes in cognitive assessment scores in relation to post-CAS flow changes, stratified by the baseline carotid stenosis group. Note: The data represent mean values of relative changes for the baseline stenosis groups. Relative changes for each participant were calculated as follows: 100 × (post-CAS value − baseline value)/baseline value. The Neurocognitive Index was derived from the computerized cognitive assessment battery, Cognitive Nervous System Vital Signs (CNSVS). Symbols indicate changes in the cognitive items, bars indicate changes in the flow, with error bars showing the standard deviation for each stenosis group. The error bar for the ipsilateral flow change and the symbol representing simple attention change in the 90% stenosis group are visually scaled down by a factor of four to allow for visualization within the *y*-axis scale.

Notably, several patterns of cognitive changes emerged across different domains in relation to post-CAS ICA flow increases and baseline stenosis severity (Figure 6). While attention and speed showed a positive response to flow increases, an opposite response was seen in memory.

## 4. Discussion

### 4.1. Summary of Main Findings

Our study found that CAS led to increased bilateral carotid artery flow. While most participants demonstrated flow increases in the treated ICA, notable contralateral flow increases were also observed in a large portion of participants. This study also suggested that CAS’s flow benefits were most substantial in participants with the most severe baseline stenosis. In comparison, flow changes following CAS were not always evident in those with less critical artery occlusion. Cognitive benefits varied across domains and were associated with flow changes, emphasizing the need for early intervention to preserve brain function.

### 4.2. Interpretation

It is well understood that CAS generally leads to increased blood flow in the treated ICA by alleviating stenosis, attributable to vessel patency restoration [20]. Our data aligned with this understanding, with most participants demonstrating moderate flow increases (of less than 36%) and higher group mean flow values post-CAS, reflecting a modest improvement in perfusion to the stented hemisphere. An extremely large increase in flow was observed in one participant, who tolerated the procedure well and demonstrated a meaningful cognitive improvement (13% increase in NCI) without any signs of hyperperfusion syndrome, suggesting a greater individual responsiveness to CAS. Longer-term follow-ups will allow for assessment of the sustainability of these effects with maintained higher-level blood flow.

Our data also confirmed previous research reporting that the degree of flow improvement depends strongly on baseline stenosis severity [21]. In our sample, changes in the ipsilateral ICA flow were the most prominent in the 90% stenosis group, which showed relative increases of up to 60%, likely due to vascular recalibration from altered blood flow resistance. In comparison, participants with 70% stenosis exhibited minimal increases or reductions in the flow, whereas those with 80% stenosis showed modest increases. These findings suggest that a more significant hemodynamic benefit may be achieved in individuals with more severe baseline ICA narrowing.

We also identified differences in CAS’s outcome related to collateral circulation. A higher proportion of our participants with isolated MCI supply showed flow increases, whereas the changes in most participants with an intact Circle of Willis were less profound. As patients with an isolated MCA are not able to receive compensatory blood flow from the other ICA or basilar supply if the flow in the ipsilateral ICA is severely stenosed, these patients, unsurprisingly, benefited the most from vascular revascularization. The flow responses varied widely, likely reflected individual differences in vascular reserve, anatomy, comorbidities, and medication use [22,23,24]. Future studies should explore how these factors interact in shaping perfusion outcomes.

Interestingly, many participants in our sample exhibited an increase in the contralateral flow. Even though, in comparison to the ipsilateral flow, the contralateral flow increases were relatively small, they were evident, especially in the 80% stenosis group and among those without an isolated MCA. Previous studies mainly reported reduced or stable contralateral flow following CAS, especially in patients with intact MCA topography. In these cases, blood flow from the contralateral artery is thought to compensate for flow reduction due to the severely narrowed ICA [16]. Upon restoration of normal flow, the reduced need for collateral support leads to decreased contralateral flow via collateral regulation. It is also possible that contralateral flow decreases reflect neural network readjustment, where reduced functional demand in the opposite hemisphere leads to lower perfusion needs. Our findings supported this theory as far as patients with intact MCAs are concerned. However, our data also suggest that the cerebrovascular system is more complex and dynamic, with regulatory mechanisms adapting to changes in vascular resistance and perfusion demands that can, in some cases, lead to increased contralateral flow. Addressing this possibility calls for further studies to verify the finding with added data to establish and test suitable hypotheses. Future research incorporating functional MRI could also help clarify how brain networks reorganize in response to restored cerebrovascular supply.

The MCA in the dominant hemisphere is responsible for the contralateral motor, sensation, and speech/language [25]. The MCA in the non-dominant hemisphere is responsible for contralateral motor, sensation, and memory. So, if there is a flow-limiting stenosis in the ICA, and the ICA supplies an isolated MCA, the neurological effects should be more pronounced because that MCA would be hypoperfused. If the ICA is recanalized, e.g., with CAS, then the neurological effects are expected to improve. The contralateral ICA flow increase contributes to the moderate increase in the total flow following CAS (even though the ipsilateral flow was the dominant driving force). Sufficient flow is a prerequisite for supplying adequate oxygen, nutrition, and energy for brain demand. We focused on MCA involvement given that its vascular territory encompasses larger brain regions critical for brain functions. MCA occlusion is typically associated with a worse prognosis for stroke. Given that infarcts in the MCA and other territories can be associated with different prognoses [13,14], future studies incorporating more detailed vascular topography can better contextualize flow and cognitive outcomes.

Although previous studies have linked global flow increases after CAS to cognitive improvements, conflicting reports across the literature have limited consensus on this relationship. In a case study by our group [4], improvements were observed in psychomotor speed, cognitive flexibility, motor speed, processing speed, and executive function following stenting. Similarly, Piegza et al. (2022) reported cognitive gains in psychomotor speed, visuospatial episodic memory, and executive function over a one-year follow-up, with no reported cognitive declines in any assessed domain [2]. Here, the various cognitive domains failed to reveal a statistically significant improvement in our sample, likely related to the small sample size, the multifactorial nature of cognitive function, and the typically limited sensitivity of standard cognitive tests to subtle changes in cerebral perfusion [16,26]. In addition, a possible delay between hemodynamic improvement and observable cognitive recovery may have contributed. For this reason, MRI studies integrating with cognitive assessments and longer-term follow-up hold the potential to further uncover associations between cerebral perfusion and cognition, along with their underlying mechanisms [27,28]. Given the overall procedural success and smooth post-intervention recovery seen in all participants, variations detected in the cognitive response are unlikely to be attributed to the intervention process per se.

Notably, despite the modest sample size, several discernible patterns emerged linking post-CAS cognitive changes to improvements in blood flow and baseline stenosis severity. Complex and simple attention showed consistent improvement with increasing ipsilateral flow across stenosis groups, suggesting that attentional domains are sensitive to perfusion and may be particularly responsive to restored flow. Motor and psychomotor speed improved as well, though with greater variability, likely reflecting a combined impact of perfusion and the structural integrity of motor-related pathways impacted by prolonged vascular compromise. Processing speed also improved in participants with 70% and 80% stenosis but plateaued or declined in the 90% group, possibly indicating a threshold beyond which chronic hypoperfusion leads to irreversible damage, limiting cognitive recovery. Perhaps by the same token, improvements in cognitive flexibility and several memory domains were predominantly observed in participants with milder baseline stenosis (70%). In contrast, visual memory showed a decline following CAS, a finding that warrants cautious interpretation and further research (e.g., task-specific variability, practice effects, or other unrelated cognitive fluctuations). Executive function and the Neurocognitive Index (NCI) both showed mixed responses across the stenosis groups, underscoring the variability in cognitive outcomes.

This variability underscores the multifactorial nature of cognitive recovery, which may be influenced not only by restored perfusion but also by pre-existing structural brain impairments, individual cognitive reserve, and the extent of vascular adaptation [29,30]. Moreover, patient-specific characteristics likely underlie the observed inter-individual variability in cognitive responses. Chronological age, baseline cognitive impairment and disease burden (for example, hypertension or diabetes), and other comorbidities can all influence cognition and postoperative brain function. Differences in overall health status, physical resilience, and cognitive reserve may further modulate recovery trajectories. Indeed, recent research demonstrated that greater preoperative frailty, quantified by cumulative health deficits, is associated with a higher risk of adverse outcomes after CAS [31]. Collectively, these results support the growing recognition that stenting may offer cognitive benefits in addition to its established role in stroke prevention. This study also highlights the importance of comprehensive patient evaluations to maximize CAS benefits by supporting early intervention, optimizing flow restoration, preventing acute and chronic complications, and preserving cognitive function—ultimately promoting wellbeing and quality of life and informing effective clinical decision-making [32,33,34].

QFlow is generally highly accurate and reproducible in large vessels like the ICA (and vertebral and basilar arteries). Several factors can potentially affect the accuracy of flow measurements. These chiefly include motion artefacts (e.g., from breathing or swallowing). Partial volume effects are especially relevant for smaller vessels when the vessel diameter is close to the voxel size (and thus can be less reliable in tortuous or narrow/distal vessels). Quantification also requires consistent ROI placement and background correction for best reproducibility. Even though absolutely accurate quantification is unrealistic, in our study, close attention was paid to optimizing the protocol by experienced MR physicists (e.g., coauthor P.S. and other MRI experts in the MRI lab). For instance, we first ensured that the MRI acquisitions were oriented as close to perpendicular as possible to straight longitudinal sections of both ICAs to limit the impact of partial volume artifacts associated with vessel-slice obliquity [35]. We also ensured that adequate temporal resolution (e.g., 15 frames of cardiac phase) and spatial resolution (e.g., more than 16 voxels within a typical internal carotid artery) were applied. We further ensured adequate depiction of the arterial flow profile in keeping with recommended practice. To account for possible background variations that may bias measurements, a 60 × 60 median filter was used for background correction [36]. The inter-reader agreement rate related to differences in the ROI delineation in flow measurements was calculated. As universally agreed upon criteria for vessel ROI definition do not exist, we opted to apply two distinct strategies, each applied by one reader, to provide an indication of the range of inter-reader variability.

### 4.3. Limitations

Several limitations of our study merit discussion. First, although our findings suggest meaningful trends, some non-significant results (*p* > 0.05) in group mean flow changes and cognitive responses clearly reflected the limited statistical power inherent to our small sample size. This constraint was further compounded by a high attrition rate—many of the initially enrolled participants withdrew due to the demanding protocol. The combined burden of MRI examination and thorough neurocognitive testing proved challenging for our cohort, as commonly seen in MRI investigations targeting cognitive decline in older patient populations. In addition to potential sample size increase consideration, future studies interested in specific cognitive aspects may improve feasibility by focusing on a targeted subset of CNSVS measures that showed promising trends. However, this would also mean sacrificing a more comprehensive understanding of certain key cognitive domains and the global cognition status.

Notably, in our study, the pulse wave monitoring with the QFlow protocol captured individualized dynamic blood pressure waves moving through the arteries. While this established approach provides physiological context on flow quantification, additional methods (e.g., ultrasound for dilation, pharmacological tests for vasoactive agents, etc.) may further help characterize the vascular tone of the carotid arteries. Additionally, in checking the flow quantification reliability, the rater who delineated ROIs based on forward-going velocity reported relatively higher flow values, although the differences were generally small. This difference was likely due to a peripheral ring of negative velocities at the vessel edge, suggesting that future studies should cautiously apply the method for improving inter-rater variability, as in our study. Furthermore, the impact of clinical considerations, such as more detailed stenosis grading and more detailed anatomical variations in the Circle of Willis and MCA, may also assist in result interpretation and deserve further analysis in future research [13]. Finally, we used a purposive clinical sample from a single health center, with all CAS procedures performed by an experienced interventional neuroradiologist. Further investigations with multi-centric studies are needed to validate our findings and improve the generalizability of the research outcomes for potential clinical application.

### 4.4. Future Directions

Despite these limitations, our study provides valuable estimates of effect size variability and the magnitude of changes, which can serve as a foundation for power calculations in developing the research line. In separate analyses, perfusion-weighted and task-based functional MRI data, which are already available with our sample, will be used to elucidate the vascular and functional mechanisms of cognitive changes and differentiate between transient post-CAS changes and sustained improvements driven by enhanced perfusion. Future research should aim to expand upon these findings by including larger, multicenter studies of more diverse cohorts, with extended follow-up periods to assess the stability of observed effects on the flow. Incorporating multimodal MRI techniques, including perfusion-weighted and functional MRI, could enhance our understanding of how vascular changes translate to cerebral perfusion, neural network reorganization, and cognitive outcomes. Stratifying patients by vascular topography (e.g., MCA supply), duration of disease, collateral flow capacity, level of frailty, and cognitive reserve may also help identify individuals most likely to benefit from CAS. Such efforts will be essential for refining individualized risk–benefit assessments, optimizing timing for intervention, and tailoring post-CAS care strategies.

### 4.5. Implications

This study contributes to the limited body of research exploring the whole-brain perfusion benefit of CAS for patients suffering from different degrees of ICA occlusion and linking global and domain-specific cognitive changes to measurable alterations in cerebral blood flow following revascularization. To our knowledge, few studies have simultaneously assessed ipsilateral and contralateral flow changes and their associations in relation to detailed cognitive responses across multiple domains. This integrated approach provides a more nuanced understanding of the relationship between vascular restoration and cognitive outcomes. The findings of this research have important implications for personalized risk stratification and treatment planning, such as identifying which patients are most likely to benefit cognitively from CAS versus those at risk of cognitive decline post-CAS (e.g., refining patient selection criteria). Furthermore, identifying patients based on cognitive responses, vascular risk burden, and collateral status may offer insight into early CAS. Knowing this may enlighten interventions tailored to patients’ pre-existing cognitive and cerebrovascular impairment, capacity, and reserve, as well as general health status. In addition, this research is relevant to post-CAS care optimization and complication minimization by developing proper cognitive training, modifiable risk factor control, and other strategies.

## 5. Conclusions

Carotid artery stenting was associated with increased cerebral blood flow, particularly in participants with the most severe baseline stenosis. Cognitive changes varied, with some domains showing improvement alongside increased flow, whereas others declined post-stenting, highlighting the complexity of neurovascular recovery and cognitive responses. Our study demonstrates the broader impact of carotid artery stenting, providing new insights to inform clinical decision-making. Ongoing analyses using perfusion and functional MRI, with extended follow-up times, will further explore the flow–cognition associations.

## Figures and Tables

**Table 1:** Demographic, health, and lifestyle characteristics of the patient participant cohort.

**Demographics**	*n*	10
	Age (years)	77.0 ± 5.6 (range: 69–86)
	Gender, male	70%
	Weight (kg)	73.9 ± 9.2 (range: 59–93)
	Height (m)	1.63 ± 0.15 (range: 1.30–1.83)
	BMI (kg/m^2^)	28.65 ± 7.83 (range: 21.46–48.52)
	Retirement Status	Retired (80%); Self-Employed (20%)
	Education	High school (70%); University (10%); Diploma (20%)
	Ethnicity	South Asian (40%); Caucasian (50%); Asian (10%)
	Marital Status	Married (70%); Single (10%); Widowed (20%)
	Living Situation	Living with family (80%); Living alone (20%)
	Handedness	Right (100%)
**Comorbidities**	Hypertension	30%
	Diabetes	40%
	Hyper-cholesterolaemia	10%
	Atrial Fibrillation	10%
	Ischemic Heart Disease	20%
	Stroke	10%
	Transient Ischemic Attack	20%
	Renal Problems	10%
**Lifestyle**	Alcohol Use	10%
	Physical Activity	No (50%); Light (20%); Moderate (30%)
	Smoking	0%
**Medication**	Blood Pressure Medications	40%
	Antiplatelet	40%
	Anti-coagulant	20%
	Other Med	40%
**Flow Measures**	With Isolated MCA	60%
	Baseline Ipsilateral Flow Rate (mL/min)	129.4 ± 48.9
	Follow-up Ipsilateral Flow Rate (mL/min)	141.9 ± 34.2
	Baseline Contralateral Flow Rate (mL/min)	177.1 ± 42.8
	Follow-up Contralateral Flow Rate (mL/min)	181.6 ± 49.9

Note: BMI, Body Mass Index; MCA, middle cerebral artery; Med, medication. Flow measurements show the group mean ± standard deviation. “*n*” represents number of participants.

**Table 2:** Individual quantitative flow and stenosis characteristics in CAS patients.

Participant ID	Isolated MCA	Ipsilateral Stenosis (%)	Baseline Ipsilateral Flow (mL/min)	First Follow-Up Ipsilateral Flow (mL/min)	Post-Stenting Ipsilateral Flow (% Change)	Contralateral Stenosis (%)	Baseline Contralateral Flow (mL/min)	First Follow-Up Contralateral Flow (mL/min)	Post-Stenting Contralateral Flow (% Change)
1	y	90	127.8	173.7	35.9	0	251.4	313.3	24.6
2	y	80	105.9	119.6	12.9	0	194.5	162.6	−16.4
3	y	95	26.2	82.8	216.4	0	186.3	181.5	−2.6
4	y	80	152.6	166.8	9.3	70	236.7	175.2	−26.0
5	y	90	101.0	93.4	−7.5	0	168.9	158.4	−6.3
6	n	90	103.2	113.7	10.2	50	126.2	136.1	7.9
7	n	80	121.2	149.6	23.5	50	120.9	160.8	33.0
8	y	80	122.9	152.1	23.8	0	130.5	221.4	69.7
9	n	80	200.8	181.5	−9.6	0	194.5	165.3	−15.0
10a	n	70	173.7	153.7	−11.5	70	184.6	188.7	2.2
10b	n	70	188.7	174.3	−7.6	0	153.7	134.0	−12.8

Note: Participants 10a and 10b represent the same individual who underwent bilateral carotid artery stenting. Each side is reported separately to reflect flow changes in both treated vessels.

## Data Availability

Data supporting the reported results may be available on request for research purposes with a formal request emailed to the corresponding authors and project principal investigators. Permission for data access for secondary analysis will be provided upon research ethics approval.

## Supplementary Materials

The following supporting information can be downloaded at https://www.mdpi.com/article/10.3390/medicina61050848/s1: Figure S1: Bland–Altman analysis of agreement in flow measurement (mL/min) between two raters, plotting the difference against the average of measurements by the raters for each artery, considering all scanning visits. The mean bias was 6.06 mL/min, with the upper and lower limits of agreement at 28.62 mL/min and −16.49 mL/min, respectively. More than 10% of the 50 datapoints fall outside the 95% CIs, suggesting a non-normal distribution of differences between the raters. Based on ½ the range between the upper and lower 95% CI, an indicator of significant change in flow of ±22% was adopted.

## Abbreviations

ACA	anterior cerebral artery
ASL	arterial spin labeling
BMI	Body Mass Index
CAS	carotid artery stenting
CEA	carotid endarterectomy
CI	confidence interval
CNSVS	CNS Vital Signs
DICE	Dice similarity coefficient
FOV	Field of View
ICA	internal carotid artery
MCA	middle cerebral artery
MRI	magnetic resonance imaging
NCI	Neurocognitive Index
ROI	region of interest

## References

[1] Youn S.W., Kim H.K., Do Y.R., Do J.K., Kwon O.C., Lee N., Lee H.J., Lee J. (2013). Haemodynamic alterations in cerebral blood vessels after carotid artery revascularisation: Quantitative analysis using 2D phase-contrast MRI. Eur. Radiol..

[2] Piegza M., Jaworska I., Piegza J., Bujak K., Dębski P., Leksowska A., Gorczyca P., Gąsior M., Pudlo R. (2022). Cognitive functions after carotid artery stenting—1-year follow-up study. J. Clin. Med..

[3] Kumar R., Chadha D., Chaddha A., Chauhan R., Singh N., Kamal P., Mishra A., Kaur N. (2022). The safety and long-term efficacy of carotid artery stenting: An all-comers registry. Cureus.

[4] Chinda B., Liang S., Siu W., Medvedev G., Song X. (2021). Functional MRI evaluation of the effect of carotid artery stenting: A case study demonstrating cognitive improvement. Acta Radiol. Open.

[5] Cremonesi A., Gieowarsingh S., Spagnolo B., Manetti R., Liso A., Furgieri A., Barattoni M.C., Ghetti L., Tavazzi L., Castriota F. (2009). Safety, efficacy and long-term durability of endovascular therapy for carotid artery disease: The tailored-carotid artery stenting experience of a single high-volume centre (tailored-CASE Registry). EuroIntervention.

[6] Elserwi A., Amer T., Soliman N., Gaballa G.M., Elmokadem A.H. (2016). Efficacy and safety of carotid artery stenting for stroke prevention. Egypt. J. Radiol. Nucl. Med..

[7] Lal B.K., Younes M., Cruz G., Kapadia I., Jamil Z., Pappas P.J. (2011). Cognitive changes after surgery vs stenting for carotid artery stenosis. J. Vasc. Surg..

[8] Lehrner J., Willfort A., Mlekusch I., Guttmann G., Minar E., Ahmadi R., Lalouschek W., Deecke L., Lang W. (2005). Neuropsychological outcome 6 months after unilateral carotid stenting. J. Clin. Exp. Neuropsychol..

[9] Tiemann L., Reidt J.H., Esposito L., Sander D., Theiss W., Poppert H. (2009). Neuropsychological sequelae of carotid angioplasty with stent placement: Correlation with ischemic lesions in diffusion weighted imaging. PLoS ONE.

[10] De Rango P., Caso V., Leys D., Paciaroni M., Lenti M., Cao P. (2008). The role of carotid artery stenting and carotid endarterectomy in cognitive performance. Stroke.

[11] Grunwald I.Q., Papanagiotou P., Reith W., Backens M., Supprian T., Politi M., Vedder V., Zercher K., Muscalla B., Haass A. (2010). Influence of carotid artery stenting on cognitive function. Neuroradiology.

[12] Xia Z.Y., Sun Q.J., Yang H., Zhang M.X., Ban R., Xu G.L., Wu Y.P., Wang L.X., Du Y.F. (2015). Effect of carotid artery stenting on cognitive function in patients with internal carotid artery stenosis and cerebral lacunar infarction: A 3-year follow-up study in China. PLoS ONE.

[13] Arboix A., Arbe G., García-Eroles L., Oliveres M., Parra O., Massons J. (2011). Infarctions in the vascular territory of the posterior cerebral artery: Clinical features in 232 patients. BMC Res. Notes.

[14] Chen B., Sun Y., Wei Z., Zhang Y. (2019). Long-term prognosis of patients with stroke associated with middle cerebral artery occlusion: Single-centre registration study. Arch. Med. Sci..

[15] North American Symptomatic Carotid Endarterectomy Trial Collaborators (1991). Beneficial effect of carotid endarterectomy in symptomatic patients with high-grade carotid stenosis. N. Engl. J. Med..

[16] Fang H., Song B., Cheng B., Wong K.S., Xu Y.M., Ho S.S.Y., Chen X.Y. (2016). Compensatory patterns of collateral flow in stroke patients with unilateral and bilateral carotid stenosis. BMC Neurol..

[17] Markl M., Frydrychowicz A., Kozerke S., Hope M., Wieben O. (2012). 4D flow MRI. J. Magn. Reson. Imaging.

[18] Bland J.M., Altman D.G. (2010). Statistical methods for assessing agreement between two methods of clinical measurement. Int. J. Nurs. Stud..

[19] Gualtieri C.T., Johnson L.G. (2008). A computerized test battery sensitive to mild and severe brain injury. Medscape J. Med..

[20] Vértes M., Nguyen D.T., Székely G., Bérczi Á., Dósa E. (2020). Middle and distal common carotid artery stenting: Long-term patency rates and risk factors for in-stent restenosis. Cardiovasc. Intervent. Radiol..

[21] Eckstein H.H., Eichbaum M., Klemm K., Doerfler A., Ringleb P., Bruckner T., Allenberg J.-R. (2003). Improvement of carotid blood flow after carotid endarterectomy—Evaluation using intraoperative ultrasound flow measurement. Eur. J. Vasc. Endovasc. Surg..

[22] Chen J.H., Wu M.H., Luo C.B., Lirng J.F., Chen S.T., Wu C.H., Guo W.-Y., Chang F.-C. (2021). Long-term imaging follow-up to evaluate restenosis in patients with carotid stenosis after angioplasty and stenting. J. Chin. Med. Assoc..

[23] Liang Z., Tang X., Chen Z. (2023). Carotid artery stenting for patients with carotid stenosis and contralateral carotid artery occlusion: A 12-year experience. Ann. Vasc. Surg..

[24] Markus H., Cullinane M. (2001). Severely impaired cerebrovascular reactivity predicts stroke and TIA risk in patients with carotid artery stenosis and occlusion. Brain.

[25] Fercho K.A., Scholl J.L., Kc B., Bosch T.J., Baugh L.A. (2023). Sensorimotor control of object manipulation following middle cerebral artery (MCA) stroke. Neuropsychologia.

[26] Gualtieri C.T., Johnson L.G. (2006). Reliability and validity of a computerized neurocognitive test battery, CNS Vital Signs. Arch. Clin. Neuropsychol..

[27] Huang D., Guo Y., Guan X., Pan L., Zhu Z., Chen Z., Dijkhuizen R.M., Duering M., Yu F., Boltze J. (2023). Recent advances in arterial spin labeling perfusion MRI in patients with vascular cognitive impairment. J. Cereb. Blood Flow. Metab..

[28] van der Thiel M., Rodriguez C., Van De Ville D., Giannakopoulos P., Haller S. (2019). Regional cerebral perfusion and cerebrovascular reactivity in elderly controls with subtle cognitive deficits. Front. Aging Neurosci..

[29] Grajauskas L.A., Guo H., D’Arcy R.C.N., Song X. (2018). Toward MRI-based whole-brain health assessment: The brain atrophy and lesion index (BALI). Aging Med..

[30] Grajauskas L.A., Siu W., Medvedev G., Guo H., D’Arcy R.C.N., Song X. (2019). MRI-based evaluation of structural degeneration in the ageing brain: Pathophysiology and assessment. Ageing Res. Rev..

[31] Liu Z., Yao Y., Zhang M., Ling Y., Yao X., Hu M. (2023). Prevalence and adverse outcomes of pre-operative frailty in patients undergoing carotid artery revascularization: A meta-analysis. Front. Cardiovasc. Med..

[32] de Lima E.P., Tanaka M., Lamas C.B., Quesada K., Detregiachi C.R.P., Araújo A.C., Guiguer E.L., Catharin V.M.C.S., de Castro M.V.M., Junior E.B. (2024). Vascular impairment, muscle atrophy, and cognitive decline: Critical age-related conditions. Biomedicines.

[33] Farooqi M.A.M., Gerstein H., Yusuf S., Leong D.P. (2020). Accumulation of deficits as a key risk factor for cardiovascular morbidity and mortality: A pooled analysis of 154 000 individuals. J. Am. Heart Assoc..

[34] Song X., Mitnitski A., Rockwood K. (2014). Age-related deficit accumulation and the risk of late-life dementia. Alzheimers Res. Ther..

[35] Pettigrew R.I., Dannels W. (1987). Use of standard gradients with compound oblique angulation for optimal quantitative MR flow imaging in oblique vessels. AJR Am. J. Roentgenol..

[36] Bernstein M.A., Zhou X.J., Polzin J.A., King K.F., Ganin A., Pelc N.J., Glover G.H. (1998). Concomitant gradient terms in phase contrast MR: Analysis and correction. Magn. Reson. Med..

