# Supplementary material for: Carotid Artery Stenting Intervention to Enhance Global Brain Blood Flow and Cognition in Carotid Artery Disease: Preliminary Findings from a Prospective Follow-Up MRI Study
Source: Medicina (Kaunas). 2025 May 5;61(5):848. doi: 10.3390/medicina61050848 (PMC12113299; doi:10.3390/medicina61050848)
Supplement: Supplementary file 1 [file medicina-61-00848-s001.zip › medicina-3595224-supplementary.pdf]

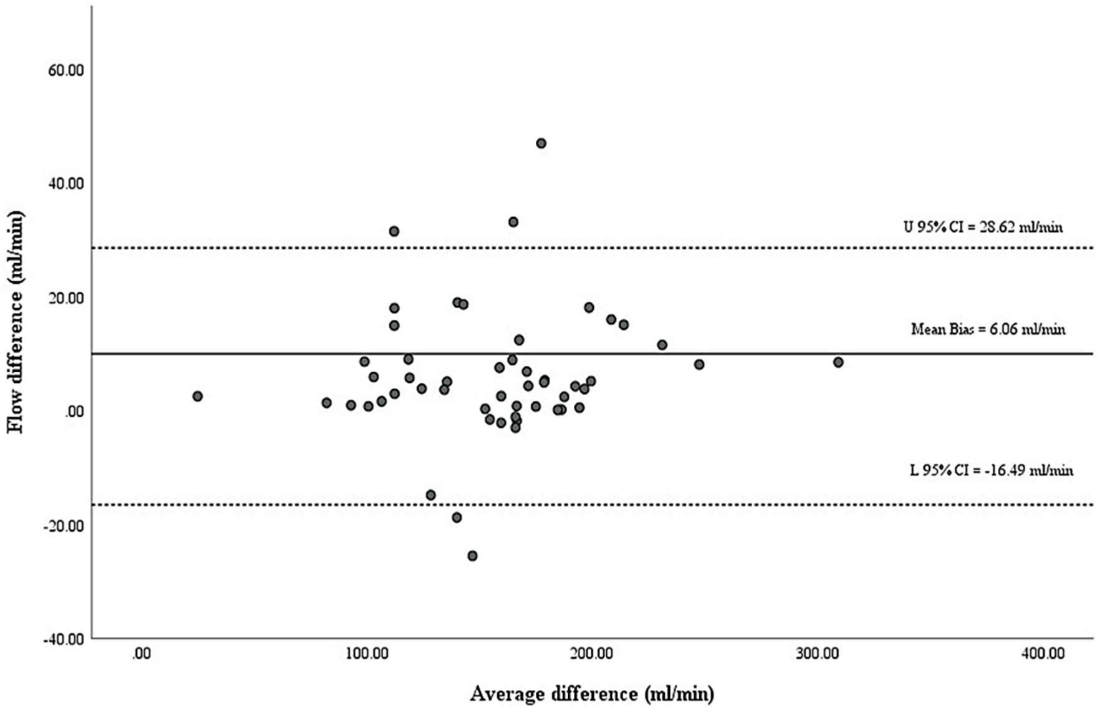

Supplementary Figure S1: Bland-Altman analysis of agreement in flow measurement (mL/min) between two raters, plotting the difference against the average of measurements by the raters for each artery, considering all scanning visits. The mean bias was 6.06 mL/min, with the upper and lower limits of agreement being at 28.62 mL/min and -16.49 mL/min, respectively. More than 10% of the 50 datapoints fall outside the 95% CIs, suggesting a non-normal distribution of difference between the raters. Based on ½ the range between the upper and lower 95% CI, an indicator of significant change in flow of +/- 22% was adopted.
